# Type I Interferon-Induced TMEM106A Blocks Attachment of EV-A71 Virus by Interacting With the Membrane Protein SCARB2

**DOI:** 10.3389/fimmu.2022.817835

**Published:** 2022-03-11

**Authors:** Xuemin Guo, Shinuan Zeng, Xiaoxin Ji, Xiaobin Meng, Nanfeng Lei, Hai Yang, Xin Mu

**Affiliations:** ^1^ Meizhou People’s Hospital, Meizhou, China; ^2^ Guangdong Provincial Key Laboratory of Precision Medicine and Clinical Translation Research of Hakka Population, Meizhou, China; ^3^ Department of Surgery, HKU-SZH & Faculty of Medicine,The University of Hong Kong, Hong Kong, Hong Kong SAR, China; ^4^ School of Pharmaceutical Science and Technology, Tianjin University, Tianjin, China; ^5^ Tianjin University and Health-Biotech United Group Joint Laboratory of Innovative Drug Development and Translational Medicine, Tianjin University, Tianjin, China

**Keywords:** interferon-stimulated gene, enterovirus A71, TMEM106A, SCARB2, antiviral activity

## Abstract

Enterovirus A71 (EV-A71) and Coxsackievirus A16 (CV-A16) are the main causative agents of hand, foot and mouth disease (HFMD) worldwide. Studies showed that EV-A71 and CV-A16 antagonize the interferon (IFN) signaling pathway; however, how IFN controls this viral infection is largely unknown. Here, we identified an IFN-stimulated gene, *Transmembrane Protein 106A* (*TMEM106A)*, encoding a protein that blocks EV-A71 and CV-A16 infection. Combined approaches measuring viral infection, gene expression, and protein interactions uncovered that TMEM106A is required for optimal IFN-mediated viral inhibition and interferes with EV-A71 binding to host cells on the receptor scavenger receptor class B member 2 (SCARB2). Our findings reveal a new mechanism contributing to the IFN-mediated defense against EV-A71 and CV-A16 infection and provide a potential strategy for HFMD treatment by using the antiviral role of TMEM106A against enterovirus.

## Introduction

Hand, foot and mouth disease (HFMD) is an early-onset disease mostly affecting children under 5 years of age. The symptoms include red rash on the hands and feet, painful red lesions on the inner cheeks and tongue, sore throat, headache, and fever (1). In rare cases, HFMD damages the central nervous system as well as the respiratory and cardiovascular systems (2), and these severe complications may be fatal (3). While more than 20 types of enterovirus can cause HFMD (4), Enterovirus A71 (EV-A71) and Coxsackievirus A16 (CV-A16), cataloged into the enterovirus-A species (5), are the most commonly reported causative agents of HFMD (6). Enteroviruses are non-enveloped, positive-sense RNA viruses (7). The binding and entry to host cells are mediated by viral capsid structure, an icosahedral viral particle composed of four structural proteins VP1, VP2, VP3, and VP4 (8). Both EV-A71 and CV-A16 viruses predominantly use host-encoded receptor scavenger receptor class B member 2 (SCARB2) or P-selectin glycoprotein ligand-1 (PSGL-1) as receptors (9). Also, CV-A10 was reported to bind to host cells *via* Kringle containing transmembrane protein 1 (KREMEN1) receptor (5). Upon receptor binding, the virion enters the cell through endocytosis (10, 11). After entry, the endosomal acidic environment facilitates the uncoating of viral capsid and the release of viral RNA into the cytoplasm. The positive-stranded RNA directs viral protein translation through the internal ribosome entry site (IRES) located within its 5′-untranslated region (5′-UTR) (12). Viral polypeptides are cleaved into functional proteins by the virus-encoded proteases 2A and 3C (13). Functional viral particles assemble with viral genomic RNAs, and the newly formed viral particles are released after the host cell is lysed (14).

Pattern recognition receptors (PRRs) are host cell-encoded proteins that sense viral infections through binding specifically to viral molecular patterns such as DNAs or RNAs and trigger downstream cascades through activating interferon (IFN) transcription. Secreted IFN proteins then serve as a signal to the host cells to launch antiviral responses, mostly through the activation of IFN-stimulated genes (ISGs) (15). Studies showed that EV-A71 infection induces IFN expression by engaging PRRs like toll-like receptor 3 (TLR3), TLR8, melanoma differentiation-associated gene 5 (MDA5), or TLR7 (16–18). To counteract IFN signaling, EV-A71 encodes proteases that disrupt or degrade key molecules (such as RIG-I, MDA5, IRF3, IRF7, IRF9, STAT1, and STAT2) in the pathway (19–23). Given that the virus targets several mediators of IFN signaling, it can be expected that IFN is detrimental to the virus and therefore is crucial for antiviral immunity. Indeed, AG129 mice lacking both type I and type II IFN are more susceptible to EV-A71 infection (24). Moreover, neutralizing antibodies against type I IFN increase the severity of the disease and the mortality rate (25). Despite the importance of IFN to control the infection, the exact mechanism of IFN-mediated inhibition of the virus remains unclear.

Transmembrane Protein 106A (TMEM106A) is a type II transmembrane protein (26). It was identified as a tumor suppressor gene in different cancer cell lines (26–28). TMEM106A was also found to express constitutively on the plasma membrane of macrophages, in which it regulates M1 polarization and pro-inflammatory functions (29, 30). Evidence regarding its antiviral activity came first from the observation that TMEM106A is an ISG in Daudi cells (B lymphoblasts) (31). Further investigation uncovered that TMEM106A restricts human immunodeficiency virus type-I (HIV-1) and other enveloped viruses by trapping viral particles from releasing (32). Similar to HIV-1-releasing inhibitory protein BST-2, the antiviral activity of TMEM106A is dependent on the plasma membrane and virion membrane (32). Whether and how TMEM106A interplays with non-enveloped viruses like EV-A71 or other enteroviruses have never been reported.

Here, we present evidence showing that TMEM106A is an inhibitory factor against EV-A71 and CV-A16 infections. Expression of TMEM106A is stimulated upon type I IFN treatment. TMEM106A specifically blocks SCARB2-mediated viral infection. This mechanistic study suggests that TMEM106A associates with SCARB2, interfering with EV-A71 binding on the host cells. Thus, our data provide a new mechanism, triggered by the IFN signaling pathway, that inhibits SCARB2-mediated enterovirus infection.

## Materials and Methods

### Cells, Plasmids, and Antibodies

Vero cell, HEK293A cell (293A in short), 293A-SCARB2 cell (293A cell stably expressing SCARB2), rhabdomyosarcoma (RD) cell, JL-1 and JL-2 mAb (fluorescein isothiocyanate (FITC)-conjugated anti-SCARB2 mAb), pCAG-DsRed (a red fluorescent protein-expressing plasmid), and EV-A71-GFP viral packaging plasmids pWSK-T7-EV71-GFP and pCDNA3.1-T7RNAP (T7 RNA polymerase), were kindly provided by Dr. Liguo Zhang, Key Laboratory of Immunity and Infection, Institute of Biophysics, Chinese Academy of Sciences (IBP, CAS). All the cells were cultured in Dulbecco’s modified Eagle’s medium (DMEM) (Invitrogen, 12800017) supplemented with 10% heat-inactivated fetal bovine serum (FBS) (Gibco), 100 U/ml penicillin, and 100 μg/ml streptomycin, at 37°C in a 5% CO2 humidified atmosphere. To generate the cell line constitutively expressing tagged TMEM106A, 293A-SCARB2 cells were transfected with pcDNA4-TMEM106A as described below and selected with Zeocin (200 μg/ml). Resistant colonies were individually expanded and validated by western blotting. One positive clone was chosen and named 293A-SCARB2-TMEM106A. This process was applied to the empty vector and resulted in control cell 293A-SCARB2-Ctrl.

The plasmid pLPCX-TMEM106A is a lentiviral-based vector expressing TMEM106A (Provided by Dr. Guangxia Gao at IBP, CAS). For the expression of myc-tagged TMEM106A full length and different truncated forms, DNAs were amplified from pLPCX-TMEM106A and cloned into pcDNA4/To/Myc-His B vector between *BamH*I and *Xba*I. To generate pcDNA3-mCherry, mCherry ORF was cloned into the pcDNA3.1 vector between *Xho*I and *Xba*I sites. pIRES2-mCherry was modified by replacing the enhanced green fluorescent protein (EGFP) coding sequence with mCherry coding sequence through cloning between *BamH*I and *Not*I sites introduced by fusion PCR. Human PSGL1 and SCARB2 cDNA were amplified from RD and 293A-SCARB2 cells, respectively, and subcloned to pIRES2-mCherry by inserting into *EcoR*I/*Sal*I, *Bgl*II/*EcoR*I sites, resulting in pPSGL1-IRES-mCherry, pSCARB2-IRES-mCherry expression vectors. All primer sequences are listed in **Table 1**.

**Table 1:** Primer Sequences.

Primer Name	Usage	Sequences (5’-3’)
mCherry-1 F	Amplification of mCherry gene	CACGGATCCGCCCCTCTCCCT
mCherry-1 R		TCCTCGCCCTTGCTCACCAT
mCherry-2 F	Fusion PCR for pIRES2-mCherry	CCTTTGAAAAACACGATGATAATATGGCCACAACCATGGTGAGCAAGGGCGAGGAGG
mCherry-2 R		CACGCGGCCGCTTTACTTGTACAGCTCGT
hPSGL1 F	Amplification of human PSGL1	CACGAATTCATGCCTCTGCAACTCCTCCT
hPSGL1 R		CACGTCGACCTAAGGGAGGAAGCTGTGCA
hSCARB2-1 F	Amplification of human SCARB2	CACAGATCTCTATGGGCCGATGCTG
hSCARB2-1 R		CACGAATTCTTAGGTTCGAATGAGGGGT
hTMEM106A-1 F	Amplification of human TMEM106A	CACGGATCCGCCACCATGGGTAAGACGTT
hTMEM106A-1 R		CACTCTAGTGGTGGGTGAGGGGTCAG
h106A (1-120) R	Together with hTMEM106A-1 F for amplification of the truncated TMEM106A a.a(1-120), a.a(1-170) and a.a(1-210), respectively	CACTCTAGACCAATGACGGACCGGGGAAA
h106A (1-170) R		CACTCTAGAACGAGGGACAGGTGCAGAAC
h106A (1-210) R		CACTCTAGAAG CCAGGTACAGATTTTGTA
h106A-C F	Together with hTMEM106A-1 R for amplification of the truncated TMEM106A-C a.a (116-262) and TMEM106A-TM-C a.a (95-262), respectively	CACGGATCCAGAGGCTGAAGCCCAAGC
h106A-TM-C F		CACGGATCCGGCAGTGGCAAGATTCCC
h106A-ΔTM F	Reverse PCR for amplification of TMEM106A-ΔTM (95-116 deleted)	AGGCTGAAGCCCAAGCACACGAAGCTC
h106A-ΔTM R		CTGGCAGGTGGGACAAGTCACGAAGC
EV-71 2C-F	qPCR for EV-71 2C	TGTATGTCTCATTATCAGGGG
EV-71 2C-R		CCACCTGTTGCTTGTAACCGT
hGAPDH-F	qPCR for human GAPDH	GAAGGTGAAGGTCGGAGT
hGAPDH-R		GAAGATGGTGATGGGATTTC
hSCARB2-2 F	qPCR for human SCARB2	GTGGGGCCATACACCTACAG
hSCARB2-2 R		GGGTCTCCAACAGATTGGTCT
hTMEM106A-2 F	qPCR for human TMEM106A	GAGAAGCAGTTGGTGGCTCT
hTMEM106A-2 R		ATCAAAGGCCACTGTGGAGG
mTMEM106A F	qPCR for monkey TMEM106A	GAGAAGCAGTTGGTGGCTCT
mTMEM106A R		ATCAAAGGCCACCGTGGAGG
mGAPDH F	qPCR for monkey GAPDH	GAAGGTGAAGGTCGGAGT
mGAPDH R		GAAGATGGTGATGGGGCTTC

The pSUPER RNAi System was used for knocking down the expression of TMEM106A. The sequence of the shRNA targeting the *TMEM106A* transcript was designed according to the recommendation of Sigma-Aldrich (https://www.sigmaaldrich.com/catalog/genes) and named 106A-shRNA. To generate pSUPER- GFP-106A-shRNA, a pair of complementary oligonucleotides 5′-GATCCCCAAGTCAATCCTGTCCTCCATTCAAGAGA*TGGAGGACAGGATTGACTT*TTTTA-3′ (sense) and 5′-AGCTTAAAAAAAGTCAATCCTGTCCTCCATCTCTTGAA*TGGAGGACAGGATTGACTT*GGG-3′ (antisense) were synthesized with 5’ ends being *Bgl*II and *Hind*III restriction site overhangs, then annealed and then cloned into the *Bgl*II and *Hind*III sites of pSUPER.retro.neo+gfp (Oligoengine, herein abbreviated for pSUPER-GFP). For each oligonucleotide, the target sequence was sense (underlined) followed by antisense orientation (Italicized) separated by a nine-nucleotide spacer.

Rabbit anti-HA tag mAb (CST, 3724S), anti-6× His tag antibody (Abcam, H8), anti-LIMP II/SCARB2 antibody (D-3) (Santacruz, sc-55570), anti-VP2 mAb (Millipore, MAB979), rabbit anti-GAPDH polyclonal antibody (Sangon Biotech, AB10016), mouse anti-β-tubulin (MG7) monoclonal antibody (Beijing Ray Antibody Biotech, RM2003), HRP-conjugated goat anti-mouse and anti-rabbit IgG antibodies (Sigma Aldrich) were used in western blotting. Alexa Fluor 555-conjugated donkey anti-Mouse IgG (H+L) and Alexa Fluor^®^ 633-conjugate donkey anti-Goat IgG (H+L) secondary antibodies were purchased from Life Technologies, and anti-SCARB2 aa27-432 (RD, AF1966), anti-SCARB2 aa339-437 (Abnova, H00000950-M01), and anti-TMEM106A (kindly provided by Dr. Yingyu Chen) were used for flow cytometry and immunofluorescence assay.

### Virus Production and Infection

EV-A71-MZ (GenBank accession no. KY582572), isolated from the throat swab of an ICU patient at Meizhou People’s Hospital (33), was propagated in RD cells. EV-A71-GFP, i.e., EV-A71 carrying an *EGFP* reporter gene inserted between 5’-UTR and VP4 gene, was established by co-transfecting pcCNA3.1-T7-RNAP and pWSK-T7-EV71-GFP plasmids at a 10:1 ratio into 293T cells (34). When GFP positive cells appeared, the supernatant was collected, added to RD cells, and incubated until apparent cytopathogenic effects (CPE) appeared. To prepare virus stocks, EV-A71-GFP viruses were propagated for one more passage in RD cells; CV-A10 and CV-A16-GZ (GenBank accession no. MG182694) viruses (provided by Dr. Weifeng Shi at Taishan Medical University and Dr. Yingxian Yin at Guangzhou Women and Children’s Medical Center, respectively) were propagated in RD cells. When CPE appeared apparently *via* microscopy observation, the supernatants were collected, aliquoted, and frozen at – 80°C for further use. Viral titers were measured by plaque assay. Virus infection was carried out as follows: cells were incubated with EV-A71 or EV-A71-GFP at a specific multiplicity of infection (MOI) in a humidified CO_2_ (5%) incubator at 37°C; one hour later, unbound viruses were washed away; cells were then cultured with fresh medium until tested. Viral titers were measured by plaque assay.

### IFN Treatment

Different kinds of cells were treated with 1000 IU/ml of recombinant human IFN-α2b (Prospec) for the indicated time, and then total RNAs were isolated and used to measure specific mRNA abundance by RT-qPCR. To examine the inhibitory effect of IFN on EV-A71, Vero, 293A-SCARB2 and RD cells were infected with EV-A71-GFP and treated with IFN-α2b at 0, 1000 and 10000 IU/ml for 8 h. The replication of the virus EV-A71-GFP was estimated by observing the GFP signal under a fluorescence microscope (System Microscope BX63, Olympus).

### Viral Plaque Assay

The viral plaque assay was performed as described previously (35). Briefly, RD or Vero cells were seeded into a 12-well plate at a density of 2x10^5^ cells/well. When reaching about 90% confluence, the cells were infected with culture supernatants containing viruses undiluted or diluted in 10-fold series for 1 h. Subsequently, the supernatants were aspirated, and cells were washed gently with PBS and then covered with DMEM containing 1% methylcellulose (Sigma-Aldrich) and 2% FBS. After incubation for 3 days, cells were fixed with 4% paraformaldehyde (Sigma-Aldrich) and stained with 0.1% crystal violet. Plaques were then quantified by visual scoring.

### Flow Cytometry (FCM)-Based Assay of GFP Production From EV-A71-GFP

To assess the effect of the TMEM106A expression on the EV71-specific receptor function, 293A cells were seeded into a 24-well plate at a density of 1.5 × 10^4^ cells/well and incubated for 18~24 h, then co-transfected with either pPSGL1-IRES-mCherry or pSCARB2-IRES-mCherry along with pcDNA4-TMEM106A or pcDNA4 vectors at a mass ratio of 1:3 using Lipofectamine 2000 (Invitrogen). After incubation for 24 h, the cells were infected with EV-A71-GFP at a specific MOI of 0.1 for 1 h and incubated for 18 h. About 1×10^6^ infected cells were collected and fixed in 4% paraformaldehyde for 15 min. After washing three times with PBS, cells were resuspended in 0.5 ml of PBS for flow cytometry (LSRFortessa, BD) assay of GFP production from EV-A71-GFP.

To estimate the effect of different TMEM106A truncated forms on EV-A71 replication, 293A-SCARB2 cells were seeded into a 24-well plate and incubated as described above, then co-transfected with the plasmids expressing full-length or truncated forms of TMEM106A together with a reporter plasmid pCAG-DsRed at a mass ratio of 3:1 and incubated for 24 h, followed by EV-A71-GFP infection at an MOI of 0.1. Twelve hours post-infection, the GFP production was measured by FCM as described above.

### Assessment of *TMEM106A* RNAi Knockdown Efficiency

293A-SCARB2 and Vero cells were transfected individually with pSUPER-GFP-106A-shRNA or pSUPER-GFP. The latter one was used as a control. After incubation for 24 h, the cells expressing high levels of GFP were sorted by FACS (BD LSRFortessa). For testing the RNAi knockdown efficiency of TMEM106A, the sorted cells were treated with IFN-α2b (1000 IU/ml) for 12 h and used for *TMEM106A* mRNA level assay by RT-qPCR, the amount of protein was assessed by western blotting, or infected with EV-A71-MZ and incubated for another 12 h for EV-A71 *2C* mRNA level assay by RT-qPCR. The GAPDH mRNA level was used as an internal control. The relative level of target mRNA in the cells expressing 106A-shRNA was normalized to the cells transfected with the control vector.

### RT-qPCR Analysis

TRIzol reagent (Invitrogen) was used for cellular RNAs extraction, and TRIzol LS reagent (Invitrogen) was used for virus RNA extraction. Extracted RNAs were treated with DNase using the RQ1 Rnase-Free Dnase Kit (Promega, M6101). cDNA was synthesized with reverse transcriptase (Takara, PrimeScript RT reagent Kit), then subjected to qPCR (Transgene, TransStart Green qPCR SuperMix) using a LightCycler 480II (Roche). The target RNA level was calculated by the comparative cycle threshold (CT) method and normalized with *GAPDH* mRNA level. The primers used for qPCR analysis are listed in **Table 1**.

### TMEM106A Overexpression in 293A-SCARB2 Cells

293A-SCARB2 cells were seeded into a 6-cm dish at a density of 10^6^ cells/well. 16-18 h later, when the cell monolayer was about 70% confluent, the cells were transfected with pcDNA4-TMEM106A-His using the Lipofectamine 2000. 48 h after transfection, the cells were transferred to a 10-cm dish and zeocin (Life technology, R25005) was added to a final concentration of 200 μg/ml to select stably transfected cell lines. This selection took from one to two weeks. The medium was carefully changed every day. The resultant cells from this line were designated 293A-SCARB2-TMEM106A.

### Viral Attachment and Entry Assay

293A-SCARB2-TMEM106A and 293A-SCARB2 cells were seeded in 24-well plates containing coverslips at a density of 10^5^ cells/well and cultured for 12 h. Then, cells were ice-chilled and incubated with EV-A71-MZ at an MOI of 100 for 1 h at 4°C, to allow viral attachment but impede viral entry. After three washes with ice-cold PBS, EV-A71 binding to the host cell surface was fixed in 250 ml ice-cold 4% paraformaldehyde for 20 min.

For entry assay, the cells were first incubated with EV-A71-MZ at 4°C for 1 h and then at 37°C for 30 min for viral entry. The cells were washed with PBS and then fixed. The fixed cells were permeabilized in PBS containing 0.2% TritonX-100 for 10 min, washed three times with PBS containing 0.1% TritonX-100 (PBST), and blocked in PBST containing 5.5% FBS for 30 min. Anti-VP2 antibody was used to detect the EV-A71 virions and then incubated with the secondary antibody Alexa Fluor 555-conjugated donkey anti-mouse IgG in the dark. Nuclei were stained with DAPI (Life Technologies). The results were analyzed by confocal microscopy (Zeiss, LSM800).

### *In Vitro* Viral RNA Production and Transfection Assay

EV-A71-GFP viral RNA was generated by using a T7 RiboMAX™ Large Scale RNA Production System-kit (Promega, P1300). The plasmid pWSK-T7-EV71-GFP was linearized by *Xho*I digestion and used as the DNA template. 293A-SCARB2-TMEM106A and 293A-SCARB2 cells were seeded in a 12-well plate at a density of 2.5 × 10^5^ cells/well one day before transfection. For the transfection, 2 μg of EV-A71-GFP RNA mixed with 2.5 μl of Lipofectamine 2000 were added to each well. The expression of GFP was monitored at regular intervals under the microscope. After 18 h, the culture supernatant was collected to measure the infectious titer of extracellular viruses by plaque assay.

### Membrane Protein Extraction and Western Blot Analysis

Total, cytoplasmic, and membrane proteins of 293A-SCARB2 and 293A-SCARB2-TMEM106A cells were individually extracted by using the ProteoExtract^®^Transmembrane Protein Extraction Kit (Novagen,71772-3), according to the manufacturer’s instructions. The protein extracts were then analyzed by western blotting using anti-SCARB2 or anti-TMEM106A antibodies to show the expression of SCARB2 and TMEM106A proteins, with the expression of GAPDH protein as a control. After that, the PVDF membrane was stained to confirm the equal loading of two samples.

### Determination of Membrane SCARB2 Conformation

293A-SCARB2-TMEM106A and 293A-SCARB2 cells were detached with trypsin, washed with PBS, and pelleted at 500g for 5 min. About 10^6^ cells were stained in 50 μl of FACS buffer (3% BSA in PBS) containing the FITC-conjugated JL-1 or JL-2 antibody (34), or common antibodies against different regions of SCARB2 (a.a27-432 and a.a339-437) for 1 h at 4°C, followed by three rounds of washing steps. After centrifugation, the cells were stained with the secondary antibodies Alexa Fluor 555-conjugated donkey anti-mouse IgG and Alexa Fluor633-conjugated donkey anti-goat at 4°C. Finally, cells were washed, resuspended in PBS, and analyzed on a cytometer FACS Calibur (C6 Acurri, USA). Unless otherwise mentioned, all experimental steps were performed on ice.

### Co-Localization of Membrane SCARB2 and TMEM106A Protein

293A-SCARB2-TMEM106A and 293A-SCARB2 cells were centrifuged, and the pellets (2 x 10^6^ cells/tube) were co-incubated with anti-SCARB2 (Abnova, H00000950-M01) and anti-TMEM106A for 1 h at 4°C, followed by incubation with corresponding FITC-conjugated secondary antibodies. The cells were then resuspended in 50 μl of PBS and transferred to coverslips in a 24-well plate, fixed using ice-cold 4% paraformaldehyde in PBS for 30 min, and incubated at room temperature for 30 min. Until the cells were fixed, all steps were performed at 4°C. The cell membrane was permeabilized, and after blocking, nuclei staining was performed. Pictures were captured as described in the viral entry assay.

### Statistical Analysis

Each figure details the statistical tests used to analyze the data set presented. Comparison of the data was performed using Student’s *t-* test by performing SPSS software. Graphpad Prism (version 8.0) was used for two-way ANOVA with Bonferroni multiple comparisons statistical analysis. Before each statistical test, the normality of each data set was assessed.

## Results

### TMEM106A Is Required for Optimal IFN-Mediated Antiviral Activity

**Figure 1:**
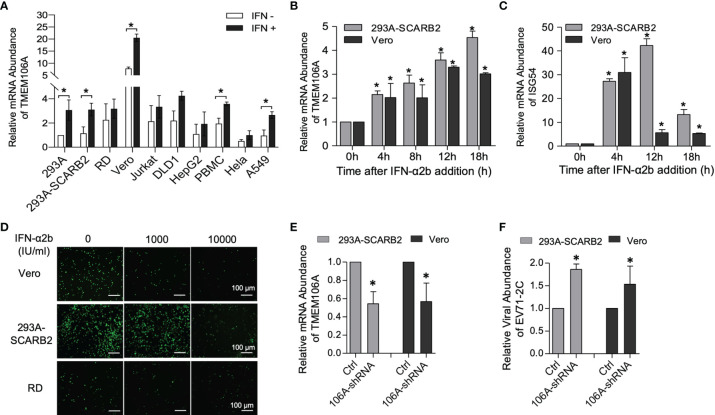
Expression and anti-EV-A71 activity of endogenous TMEM106A. **(A)** RT-qPCR assessment of endogenous TMEM106A mRNA level in different cell lines with or without IFN-α2b (1000 IU/ml) treatment for 18 h. The mRNA level of *TMEM106A* was normalized to that of *GAPDH* in the different RNA samples. The normalized data in 293A cell line was set as 1. Statistical differences between IFN-α2b treatment and mock treated were assessed using Student’s *t* test. **(B)** Cells from the 293A-SCARB2 or Vero lines were stimulated with IFN-α2b (1000 IU/ml) for 0 h, 4 h, 8 h, 12 h and 18 h, and assessed for *TMEM106A* RNA expression by RT-qPCR. *TMEM106A* mRNA level was normalized according to *GAPDH* mRNA level. The results are expressed as fold change relatively to the levels in unstimulated cells (0 h) set as 1. Statistical differences between IFN-α2b treatment and mock treated were assessed using Student’s *t* test. **(C)** Cells from the 293A-SCARB2 or Vero lines were stimulated with IFN-α2b as in **(B)** and assessed for *ISG54* mRNA level by RT-qPCR. Statistical differences between IFN-α2b treatment and mock treated were assessed using Student’s t test. **(D)** Assessment of the antiviral activity of IFN-α2b against EV-A71-GFP in Vero, 293A-SCARB2, and RD cells. 293A-SCARB2, Vero, and RD cells were infected with EV-A71-GFP (MOI=0.1) and then treated with IFN-α2b at 1000 or 10000 IU/ml, with untreated as control. After 8 h incubation, viral infection was examined by the expression of GFP. **(E)** *TMEME106A* mRNA knockdown in 293A-SCARB2 and Vero cells was achieved by transfection with the shRNA-encoding plasmid pSUPER-GFP-TMEM106A-shRNA. Knockdown efficiency, compared with the cells transfected with the control plasmid pSUPER-GFP (Ctrl), was assessed by RT-qPCR. Twenty-four hours post-transfection, the GFP-positive cells were FACS-sorted and treated with IFN-α2b for another 12 h. *TMEM106A* mRNA level was measured by RT-qPCR, with GAPDH mRNA level as internal control. Statistical differences between control and shRNA-transfected cells were assessed using Student’s *t* test. **(F)** EV-A71 *2C* mRNA level in the cells where TMEM106A expression was knocked down by shRNA. The GFP-positive cells sorted 24 h after transfection and stimulated with IFN-α2b for 12 h were infected with EV-A71 (MOI=0.1). Twelve hours post-infection, viral RNA level was quantified by RT-qPCR with GAPDH mRNA level as internal control. Statistical differences between control and shRNA-transfected cells were assessed using Student’s *t* test. All the RT-qPCR results above are presented as mean ± SD of three independent experiments. *p < 0.05.

The level of endogenous *TMEM106A* mRNA was assessed in commonly used cell lines as well as peripheral blood mononuclear cells (PBMC) with or without IFN-α2b treatment. Among the tested cells, Vero cells produced the highest level of *TMEM106A* mRNA, whereas all other cells produced similar levels (**Figure 1A**), including the 293A-SCARB2 cell line stably expressing a *SCARB2*-transgene, suggesting that the ectopic expression of SCARB2 did not change the *TMEM106A* RNA level. Consistent with previous studies showing that *TMEM106A* is an ISG in Daudi cells (31), IFN-α2b treatment enhanced *TMEM106A* expression (**Figure 1A**). Upon the stimulation of IFN-α2b, the expression of endogenous TMEM106A increased variably in different cell lines (**Figure 1A**). In both 293A-SCARB2 and Vero cells, this induction was in a time-dependent manner (**Figure 1B**). This upregulation was weaker than that of *ISG54* (**Figure 1C**) but showed a more persistent pattern.

Next, we asked whether type I IFN treatment could inhibit EV-A71 infection. Albeit the virus encodes mechanisms that antagonize IFN signaling, IFN-α2b treatment restricted EV-A71 infection in a dose-dependent manner in Vero, 293A-SCARB2 and RD cells (**Figure 1D**). To determine if TMEM106A contributed to this restriction, shRNAs against *TMEM106A* (106A-shRNA) were introduced into 293A-SCARB2 and Vero cells and collected by FACS sorting. All sorted cells were treated with IFN-α2b. About half of the *TMEM106A* RNAs were knocked down by shRNAs (**Figure 1E**), and this almost doubled the viral abundance (**Figure 1F**). This result was apparent in both 293A-SCARB2 cells and Vero cells. Thus, *TMEM106A* is an ISG and is required for optimal IFN-mediated EV-A71 restriction.

HEK293 cell line lacks the expression of TLRs in normal states (36), making it an ideal cell line for studying the function of an ISG. Compared to 293A alone, 293A-SCARB2 enhanced the infection of EV-A71 significantly (37). We also observed this phenomenon (data not shown). To facilitate the infection, we used 293A-SCARB2 for the next assays.

### TMEME106A Inhibits SCARB2-Mediated Virus Binding and Infection

**Figure 2:**
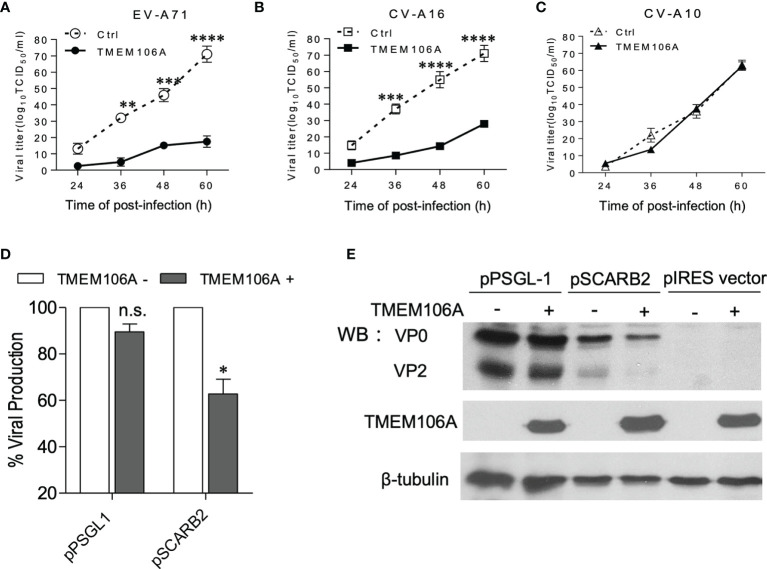
Antiviral activity in 293A-SCARB2-TMEM106A cells. **(A–C)** 293A cells stably expressing SCARB2 alone (Ctrl) or SCARB2 together with TMEM106A (TMEM106A) were infected with EV-A71-MZ **(A)**, CV-A16-GZ **(B),** or CV-A10 **(C)** at an MOI of 0.01. The viral yields were determined at different times post-infection by a TCID50 assay. The data are mean ± SD of three independent experiments. Statistical differences were assessed using a two-way ANOVA with Bonferroni multiple comparisons and are highlighted by * (p < 0.05), ** (p < 0.005), *** (p < 0.0005) and **** (p < 0.0001). **(D, E)** 293A cells co-transfected with 100 ng of either pPSGL1-IRES-mCherry or pSCARB2-IRES-mCherry, and with 300 ng of pcDNA4-TMEM106A or pcDNA4 vectors at a mass ratio of 1:3 for 24 h. Then the cells were infected with EV-A71-EGFP at an MOI of 0.1 for 18 h. Half of the cells were analyzed by FACS **(D)**, and the other half was analyzed by western blotting **(E)**. VP0 (VP2+VP4) and VP2 are viral proteins. β-tubulin serves as loading control. Statistical differences between control and pcDNA4-TMEM106A-transfected cells in PSGL-1- or SCARB2-expressing group were assessed using Student’s *t* test. Data are means ± SD of three independent experiments. *p < 0.05; n.s., no statistical significance.

To further investigate the inhibitory mechanism operated by TMEM106A on viral infection, 293A-SCARB2-TMEM106A, a cell line ectopically expressing TMEM106A, was generated and infected with three types of enteroviruses: EV-A71, CV-A16 or CV-A10, with 293A-SCARB2-Ctrl expressing a corresponding empty vector as control. Virus-containing culture supernatants were collected at different time points post infection and their titers were measured. Data showed that in TMEM106A-expressing cells, EV-A71 titer was dramatically decreased compared to that in control cells (**Figure 2A**). Similarly, CV-A16 replication was reduced in the presence of TMEM106A (**Figure 2B**). In contrast, CV-A10 infection was not affected by TMEM106A expression (**Figure 2C**). Thus, TMEM106A inhibits both EV-A71 and CV-A16 infection, but not CV-A10.

Although CV-A10 belongs to the enterovirus family, it uses host cell expressed KREMEN1 as the receptor, contrary to EV-A71 and CV-A16, which use SCARB2 or PSGL1 (5). Our observation suggested that TMEM106A-mediated viral inhibition may be receptor-dependent. To test this hypothesis, 293A cells were co-transfected with plasmids expressing different receptors and TMEM106A, which were subsequently challenged by *EGFP*-encoding EV-A71 infection. Viral infection was measured by flow cytometry. Only SCARB2-expressing cells showed a decrease of viral infection, not PSGL1-expressing cells (**Figure 2D**). We also tested levels of viral protein VP0 (the precursor of the capsid proteins VP2 and VP4) and VP2. Consistently, the TMEM106A-dependent decrease of viral protein expression was only observed in SCARB2-expressing cells, but not PSGL-1-expressing cells (**Figure 2E**). Thus, our data indicated that TMEM106A blocks SCARB2-dependent viral infection.

### TMEM106A Blocks Virus Binding to Target Cells

**Figure 3:**
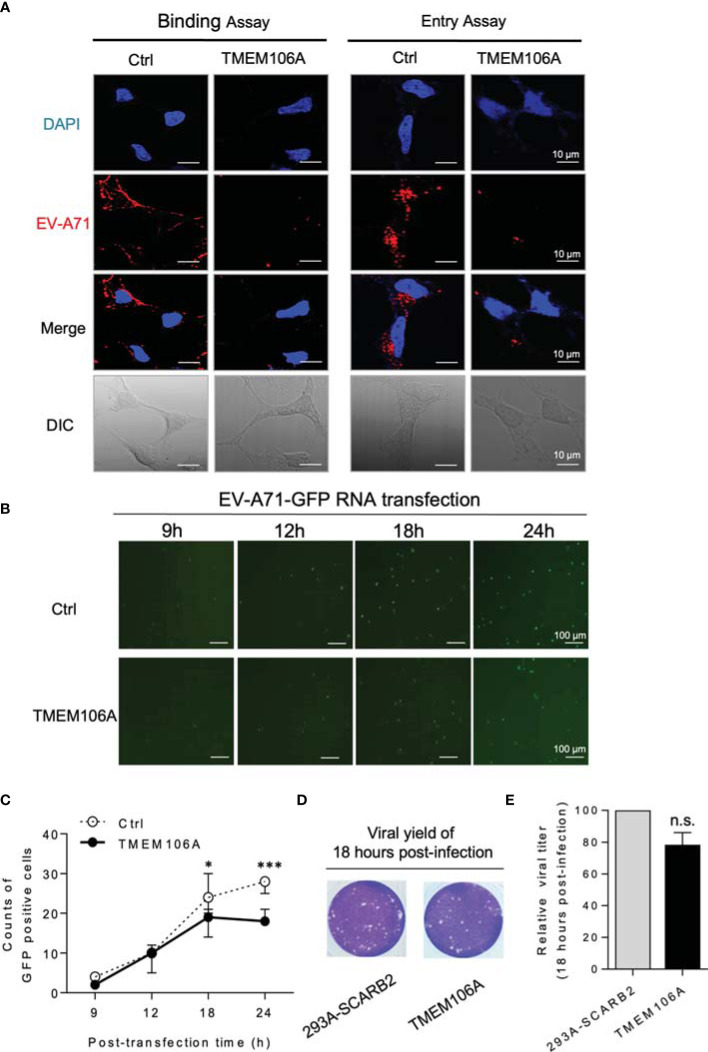
Mechanistic study of the inhibition of EV-A71 by TMEM106A. **(A)** The effect of TMEM106A expression on the attachment (left) and endocytosis (right) of EV-A71 was examined by incubating control cells (293A-SCRB2) and cells expressing TMEM106A (293A-SCRB2-TMEM106A) with EV-A71-MZ (MOI=100) for 1 h at 4°C (binding), with or without an additional 30-min incubation at 37°C (endocytosis). The EV-A71 virions were detected by immunofluorescence assay using a primary antibody against EV-A71 VP2 and an Alexa-Fluor 555-conjugated secondary antibody. The cells were counterstained with DAPI to show the nucleus. Bar=10 μm. DIC, differential interference contrast. **(B)** To examine the effect of TMEM106A expression on the post-entry stages of EV-A71 replication, EV-A71-EGFP RNAs were transcribed from the linearized pWSK-EV71-EGFP, and then transfected into 293A-SCARB2-TMEM106A or control 293A-SCARB2 cells. The expression of EGFP and the cellular viral RNA levels at different time points post transfection were examined by fluorescence microscope. **(C)** The GFP-positive cells in 50 fields were counted and the mean number was calculated. Statistical differences between Ctrl and TMEM106A were assessed using Student’s *t* test. * (p < 0.05) and *** (p < 0.001). **(D)** Eighteen hours after transfection with the viral RNA, the viral yield in 293A-SCARB2-TMEM106A and 293A-SCARB2 cells was measured by plaque assay. **(E)** The value from the 293A-SCARB2 control cells was set at the 100% of virus production to estimate remaining percentage of viral production in presence of TMEM106A. Statistical differences between control and TMEM106A were assessed using Student’s *t* test. The data are mean ± SD of three independent experiments. n.s., no statistical significance.

Next, we set out to determine if SCARB2-mediated viral binding and entry was blocked by TMEM106A. EV-A71 binds to the cell surface receptor and enters the cell through the endocytosis provoked by engagement of the receptor. Experimentally, endocytosis can be blocked by lowering the culture temperature, while the virus-receptor binding remains effective. TMEM106A-expressing 293A-SCARB2 cells, or controls harboring an empty vector, were first incubated with EV-A71 at 4°C for attachment, and then at 37°C to allow the entry of the virus. This protocol can separate the steps of viral binding to host cell and entry. Cells taken after the first condition or after both conditions were stained with DAPI and fluorescent antibodies against the EV-A71 capsid protein VP2. This experiment showed that in the presence of TMEM106A, EV-A71 binding to host cells was dramatically decreased, the entry of viral particles was consequently largely reduced (**Figure 3A**).

To further investigate if TMEM106A affected other steps of the viral life cycle, EV-A71-GFP RNA was *in vitro* transcribed using pWSK-EV71-GFP as a template and transfected cells, allowing skipping the receptor binding and entry steps. TMEM106A expression did not affect viral GFP signals at 9 or 12 h post transfection, and slightly decreased GFP expression at 24 h post transfection (**Figures 3B, C**). We deduced that the slight inhibition at 24 h was due to TMEM106A blocking new cycles of EV-A71 virion infection, occurring after EV-A71 RNA transfection. At 18 h post transfection, the culture supernatants containing the virus were collected and used to infect recipient cells. This experiment indicated that viral titers from TMEM106A empty and expressed cells were similar at this time point (**Figures 3D, E**). Altogether, these data suggest that TMEM106A inhibits EV-A71 by blocking its binding to the entry receptor SCARB2 on host cells.

### TMEM106A Colocalizes With SCARB2 and Produces Steric Hindrance on SCARB2

**Figure 4:**
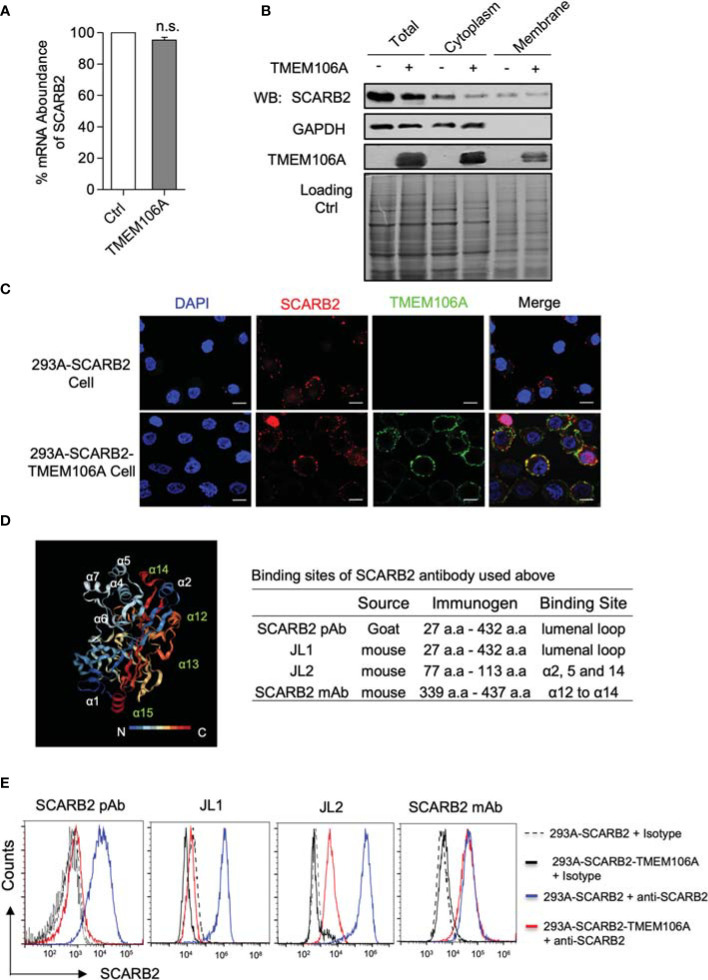
SCARB2 expression and co-localization with TMEM106A, and mapping of SCARB2/TMEM106A association. **(A)** Assessment of *SCARB2* mRNA level in the presence of over-expressed TMEM106A protein. Total RNAs were isolated from 293A-SCARB2-TMEM106A and 293A-SCARB2 control cells and subjected to RT-qPCR assay. The mRNA level of *SCARB2* was normalized to that of *GAPDH*. The relative mRNA level of control cells was set as 100%. Statistical differences between Ctrl and TMEM106A were assessed using Student’s *t* test. The data are mean ± SD of three independent experiments. n.s., no statistical significance. **(B)** Assessment of SCARB2 protein level in the presence of over-expressed TMEM106A protein. Total, cytoplasmic and membrane protein extracts were prepared from 293A-SCARB2-TMEM106A and 293A-SCARB2 control cells. Western blot analysis was carried out by using anti-SCARB2 and anti-TMEMA106A antibody, respectively, to compare the protein levels of SCARB2 as well as TMEM106A. The membrane was then stained to confirm the equal loading of two samples. **(C)** Immunofluorescence microscopic picture showing the membrane co-localization of SCARB2 and TMEM106A. 293A-SCARB2-TMEM106A and 293A-SCARB2 cells were collected and co-incubated with anti-SCARB2 and anti-TMEM106A antibodies, followed by incubation with corresponding FITC-conjugated secondary antibodies. The cells were then transferred to coverslips and fixed with ice-cold 4% paraformaldehyde. Finally, the cell membrane was permeabilized, and nuclear DNA was stained with DAPI (blue). Bar=10 μm. **(D)** Crystal structure of SCARB2 ectodomain (PDB:4TW2) (left panel) featuring the binding sites of the antibodies recognizing different domains of SCARB2 (right panel). **(E)** Conformation analysis of membrane SCARB2 *via* surface staining analyzed by flow cytometry. 293A-SCARB2-TMEM106A and 293A-SCARB2 cells were collected, incubated with the FITC-conjugated JL-1 or JL-2 antibody, or common antibodies against different regions of SCARB2 (a.a. 27-432 and a.a. 339-437) for 1 h at 4°C, followed by the Alexa Fluor 555-conjugated anti-mouse IgG and Alexa Fluor633-conjugated anti-goat IgG staining. Finally, the cells were analyzed by using flow cytometry. Control 293A-SCARB2 and 293A-SCARB2-TMEM106A cells were incubated with isotype (solid and dashed in black) as florescence negative control. Blue and red lines represent cells incubated with the indicated SCARB2 antibodies.

Next, we investigated how TMEM106A would affect SCARB2-mediated viral binding by using the 293A-SCARB2-TMEM106A cell line, with the 293A-SCARB2 cell line as a control. We first asked whether TMEM106A would interfere with SCARB2 expression. Equal number of 293A-SCARB2-TMEM106A and 293A-SCARB2 cells were collected and used for total RNAs isolation. Meanwhile, the total cell lysates, cytoplasmic and membrane proteins were prepared individually. RT-qPCR results indicated that the expression of TMEM106A did not change the mRNA level of *SCARB2* (**Figure 4A**). Western blotting also showed that TMEM106A expression had little effect on SCARB2 abundance at the cell membrane (**Figure 4B**). More importantly, TMEM106A is also localized to the cell membrane (**Figure 4B**). The localization of TMEM106A and SCARB2 at the membrane raised the hypothesis that the two proteins may associate with each other, which in turn, sterically block viral binding to SCARB2. Indeed, immunofluorescent staining indicated that SCARB2 colocalized with TMEM106A on the plasma membrane of the cells (**Figure 4C**), suggesting their association.

To map the affected sites, we used several anti-SCARB2 antibodies targeting different epitopes of the protein. The structural analysis of SCARB2 showed several helices inside the lumenal loop exposed to the extracellular environment. Previous studies suggested that this region is critical for EV-A71 recognition and binding by VP1 and VP2 (38). Polyclonal (SCARB2 pAb) and JL1 antibodies recognize the entire lumenal loop, JL2 antibody targets the alpha helices 2, 5 and 14, and the monoclonal antibody (SCARB2 mAb) binds to alpha helices 12 and 14 (**Figure 4D**, right panel). Cytometric analysis showed that, while all four antibodies recognized SCARB2 successfully in the absence of TMEM106A, only SCARB2-mAb still bound SCARB2 in the presence of TMEM106A. The other three antibodies were blocked by the presence of TMEM106A (**Figure 4E**). These data suggested that TMEM106A associates with the SCARB2 lumenal loop, including alpha helices 2, 5 and 14, which are critical sites for EV-A71 binding (38). Taken together, our data suggest that through colocalizing to the SCARB2 proteins, TMEM106A provokes a steric hindrance for EV-A71 binding.

### TMEM106A Functions Through Its Extracellular Region

**Figure 5:**
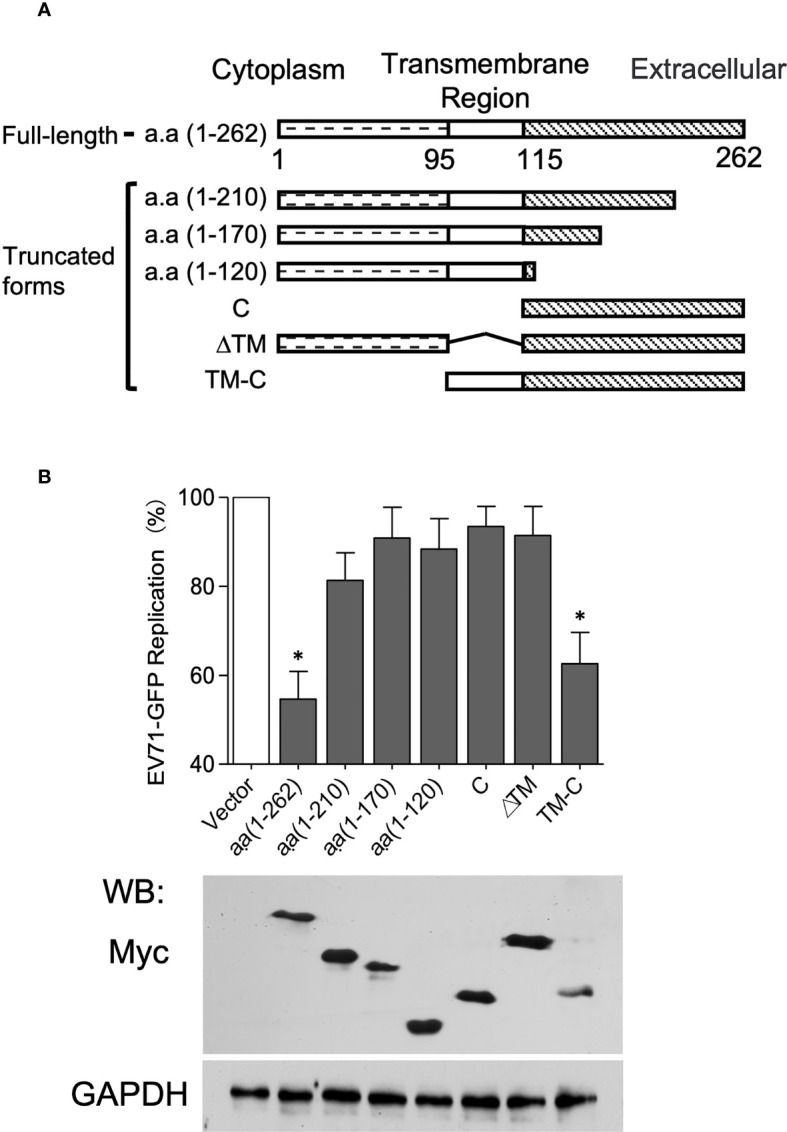
The C-terminal domain of TMEM106A is involved in EV-A71 attachment. **(A)** Domain architecture of TMEM106A protein and truncations. **(B)** Antiviral activity of different deletion mutants of TMEM106A. Plasmids expressing myc-tagged wild-type (a.a. 1-262) or truncated TMEM106A were individually transfected into 293A-SCARB2 cells together with a reporter plasmid pCAG-DsRed at a ratio of 3:1, followed by EV-A71-GFP infection at an MOI of 0.1. An empty vector without *TMEM106A* fragment was used as control. Level of full-length TMEM106A or deletion mutants were analyzed by western blotting using an anti-myc mAb (lower panel). The GFP signal produced by EV-A71-GFP was detected by FACS. The data were normalized according to the GFP produced in the control cells transfected with the empty vector, in which where none of the TMEM106A forms were expressed, set as 100% (upper panel). The results are represented as mean ± SD obtained in three independent experiments. *p < 0.05.

TMEM106A is a type-II membrane protein, containing a cytoplasmic region (amino acids 1-95, a.a 1-95), a transmembrane region (a.a 96-115) and an extracellular region (amino acids 116-262) (**Figure 5A**) (39). We reasoned that the extracellular region of TMEM106A could be responsible for SCARB2 association and subsequent EV-A71-binding inhibition. To test this hypothesis, we constructed vectors expressing different truncated forms of TMEM106A (**Figure 5A**). Expression level and size of all truncated and full-length proteins were verified by western blotting on whole cell lysates (**Figure 5B**). TMEM106A proteins lacking the C-terminal region (a.a 1-120, a.a 1-170 and a.a 1-210) did not inhibit EV-A71 replication, as monitored by virus-encoded GFP expression, whereas the transmembrane region together with the extracellular region (TM-C) retained a comparable antiviral activity as the full-length protein (a.a 1-262) (**Figure 5B**). The extracellular region alone (C) was unable to inhibit EV-A71 replication, likely because it lacked the membrane targeting signal sequence for correct localization. These data suggested that TMEM106A locates on the cell membrane and blocks EV-A71 infection through its extracellular region anchored on the plasma membrane, interfering with the virus-binding site on SCARB2.

## Discussion

**Figure 6:**
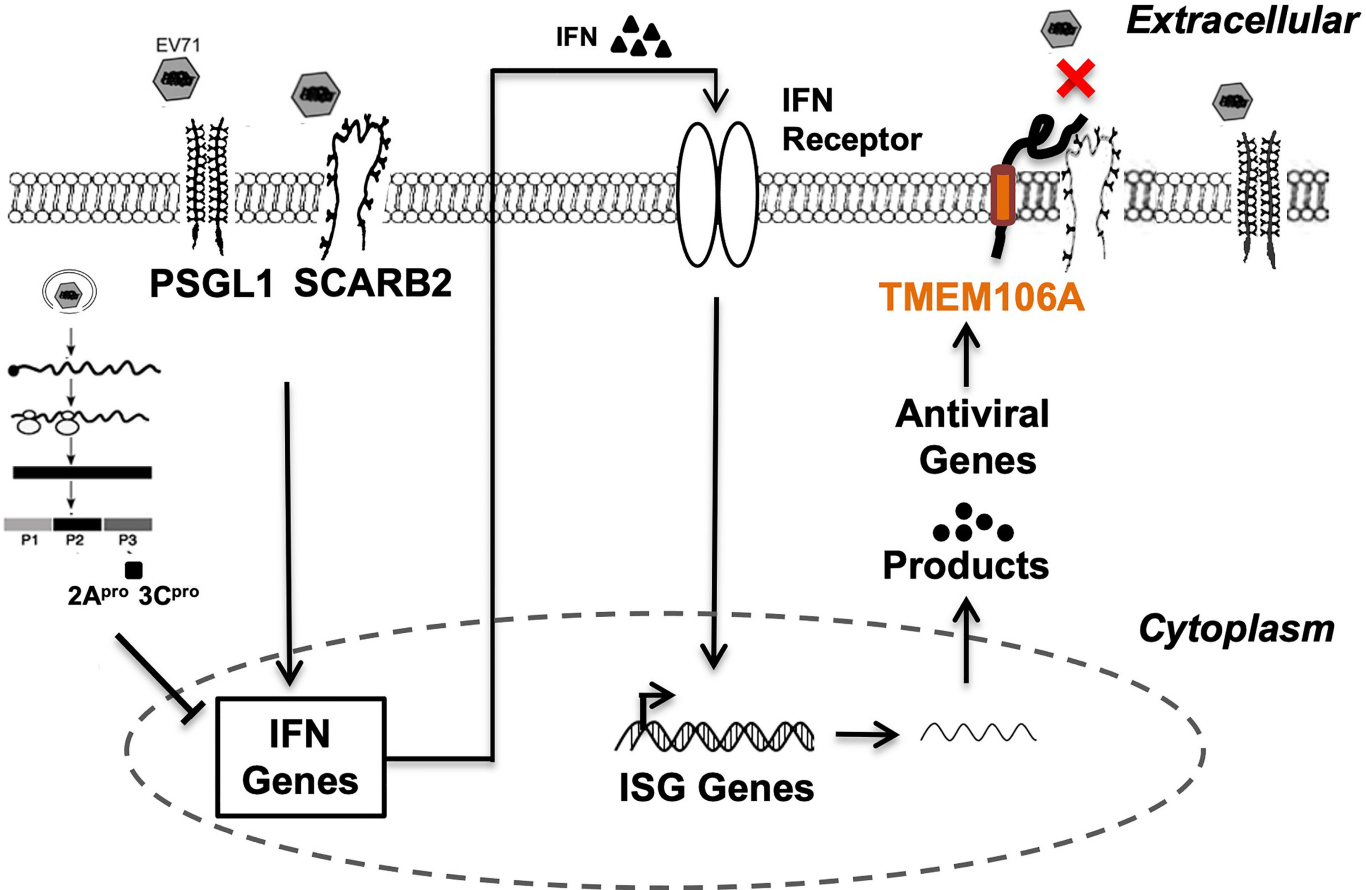
Proposed mechanism of the suppression of EV-A71 infection by TMEM106A. Both SCARB2 and PSGL-1 act as cell surface receptors enabling EV-A71 infection. SCARB2 facilitates EV-A71 infection by playing critical roles in virus attachment, entry and uncoating. Innate immune response to viral infection, through engagement of receptors, activates signaling pathways that induce the expression and secretion of type I IFN. In turn, IFN molecules bind their cognate receptors, signal through the JAK-STAT pathway, and induce hundreds of ISGs. One of them, *TMEM106A*, encodes a transmembrane protein, which exerts antiviral activity by blocking the recognition site of EV-A71 through its association with SCARB2, but not PSGL-1.

Here, we showed that TMEM106A is an ISG upregulated upon type I interferon treatment and is required for optimal IFN-mediated antiviral activity against EV-A71 infection (**Figure 1**). TMEM106A blocks SCARB2-mediated EV-A71 and CV-A16 infection but does not affect infections mediated by other receptors (**Figure 2**). Further, we showed that TMEM106A specifically targets the SCARB2 lumenal loop, creating a steric hindrance for EV-A71 binding (**Figures 3** and **4**). The transmembrane region and extracellular region of TMEM106A are responsible for this competition (**Figure 5**). Based on these results, we hypothesized a working model for the inhibition of EV-A71 infection by TMEM106A (**Figure 6**).

The antiviral role of IFN is mostly mediated by the induction of ISG expression. Many studies have addressed the mechanisms whereby EV-A71 antagonizes IFN signaling (19–23); however, how ISGs inhibit EV-A71 infection remains elusive. We found that *TMEM106A* is not a robustly stimulated ISG compared to *ISG54* but is more durable (**Figures 1B, C**). Our results (**Figures 1E, F**) suggested that TMEM106A could act as a host factor protecting host cells from EV-A71 infection. Recently, TMEM106A was found to inhibit HIV-1 and other enveloped virus release (32). It is incorporated into progeny virions and is located on the virion membrane. The incorporated TMEM106A then interacts with other TMEM106A proteins on the plasma membrane through intermolecular interactions between the two extracellular domains, which in turn, forces the progeny virion to attach to the cell surface (32). We observed that in the case of non-enveloped enteroviruses, TMEM106A selectively inhibited EV-A71 and CV-A16, but not CV-A10, in a receptor-dependent manner, suggesting a new antiviral mechanism. Further exploring how TMEM106A impacts other types of enteroviruses will help to establish its role in antiviral response. How this influences enterovirus infection *in vivo* is also of interest to investigate in the future.

It was reported that TMEM106A is conserved among species including humans, chimpanzees, rhesus macaque, dogs, cows, mice, and rats (26), implicating it plays important roles *in vivo*. TMEM106A was initially identified as a tumor suppressor gene, down-regulated in expression in gastric cancer (GC) cell lines but not in normal gastric tissues (26). The regulatory role of constitutively expressed and lipopolysaccharide (LPS)-induced TMEM106A on the immunological activity of macrophage *via* the MAPK and NK-κB signaling pathways were also reported (29, 30). All these findings suggested that TMEM106A may link the extracellular environment to intracellular responses. Our mapping assays showed that the extracellular region of TMEM106A anchored on the plasma membrane is sufficient to inhibit virus infection (**Figure 5**). How this TMEM106A-SCARB2 association affects macrophage needs further study in the future.

SCARB2 is a type-III membrane protein, bearing a 400 a.a luminal domain in the extracellular region. Its physiological function consists of mediating the transport and reorganization of the endosomal/lysosomal compartment’s membrane (40). The alpha 5 and 7 helices of SCARB2 are known as key regions for EV-A71 binding (38). We observed that colocalization of TMEM106A with SCARB2 blocks the accessibility of antibodies targeting the luminal domain. More importantly, antibodies directed against regions of helices 2, 5 and 14 could not bind SCARB2 in the presence of TMEM106A, suggesting that these regions are occupied by TMEM106A (**Figures 4D, E**). Correct membrane-anchoring of the extracellular region of TMEM106A is required for its antiviral activity (**Figure 5B**), highlighting the importance of the positioning of TMEM106A and SCARB2 for binding. Whether this mode of action represents a common antiviral mechanism among species is of broad interest to tackle viral infections. Targeting SCARB2-virus binding is promised to play an important role in restricting viral infection and spread. Our findings revealed a potential tool for EV-A71 prevention by utilizing the TMEM106A-SCARB2 interaction.

## Data Availability Statement

The datasets presented in this study can be found in online repositories. The names of the repository/repositories and accession number(s) can be found in the article/**Supplementary Material**.

## Ethics Statement

The Ethics Committee of Meizhou People’s Hospital waived the requirement for ethical approval and written informed consent for participants in this study because this application involves the use of de-identified swabs that could not be identified, in accordance with the national legislation and the institutional requirements.

## Author Contributions

XG, XM, and SZ were major contributors in writing this manuscript. XG, SZ, and XM designed the experiments. XG, SZ, XBM, NL, XJ, and HY carried out the experiments and data collection. XG, SZ, and XBM performed the data analysis. All authors read and approved the final manuscript.

## Funding

This work was supported by the grants from the Natural Science Foundation of Guangdong Province (2019A1515012133), the Guangdong Provincial Key Laboratory of Precision Medicine and Clinical Translation Research of Hakka Population (2018B030322003), the Science and Technology Program of Meizhou (2019B0202001, 2019B001), Key Scientific and Technological Project of Meizhou People’s Hospital (PY-A2019003).

## Conflict of Interest

The authors declare that the research was conducted in the absence of any commercial or financial relationships that could be construed as a potential conflict of interest.

## Publisher’s Note

All claims expressed in this article are solely those of the authors and do not necessarily represent those of their affiliated organizations, or those of the publisher, the editors and the reviewers. Any product that may be evaluated in this article, or claim that may be made by its manufacturer, is not guaranteed or endorsed by the publisher.

## Abbreviations

EV-A71, Enterovirus A71; CV-A16, Coxsackievirus A16; CV-A10, Coxsackievirus A10; HFMD, hand, foot and mouth disease; IFN, interferon; TMEM106A, transmembrane protein 106A; SCARB2, scavenger receptor class B, member 2; PSGL-1, P-selectin glycoprotein ligand-1; KREMEN1, Kringle Containing Transmembrane Protein 1; IRES, internal ribosome entry site; 5′-UTR, 5′-untranslated region; PRR, pattern recognition receptor; ISG, interferon-stimulated gene; TLR, toll-like receptor; MDA5, melanoma differentiation-associated gene 5; HIV-1, human immunodeficiency virus type I; RD, rhabdomyosarcoma; FITC, fluorescein isothiocyanate; T7RNAP, T7 RNA polymerase; DMEM, Dulbecco’s modified Eagle’s medium; FBS, fetal bovine serum; EGFP, enhanced green fluorescent protein; CPE, cytopathogenic effect; MOI, multiplicity of infection; PBMC, peripheral blood mononuclear cell; a.a, amino acid; TM, transmembrane; GC, gastric cancer; LPS, lipopolysaccharide.
